# Design and Construction of a Fusion Peptide Containing B1, B2, B4, and EPC1 Epitopes for Diagnosis of Human Cystic Echinococcosis

**Published:** 2019-09

**Authors:** Enayat DARABI, Elahe MOTEVASELI, Mohammad Reza KHORRAMIZADEH, Mehdi MOHEBALI, Mohammad Bagher ROKNI, Farzaneh ZAHABIUN, Eshrat Beigom KIA

**Affiliations:** 1. Department of Medical Parasitology and Mycology, School of Public Health, Tehran University of Medical Sciences, Tehran, Iran; 2. Department of Molecular Medicine, School of Advanced Technologies in Medicine, Tehran University of Medical Sciences, Tehran, Iran; 3. Biosensor Research Center, Endocrinology and Metabolism Molecular-Cellular Sciences Institute, Tehran University of Medical Sciences, Tehran, Iran

**Keywords:** Cystic echinococcosis, Diagnosis, Insilico, B cell epitope, Fusion epitope, Antigen B

## Abstract

**Background:**

Cystic echinococcosis (CE), larval stage of *Echinococcus granulosus*, immunodiagnostics is still a challenge due to asymptomatic nature of CE during the early phase of infection and imperfection of diagnostic antigens. In silico design and assessments of hydatid cyst antigens provide preeminent information for novel and favorable diagnostic methods.

**Methods:**

This study was performed at the Tehran University of Medical Sciences, Tehran, Iran in 2018. The sequences of B2, EPC1, B1 and B4 antigens were collected and analyzed for sequence conservancy by protein BLAST search and CLUSTALW multiple sequence alignment. The secondary and 3D structures were predicted using ab initio and threading methods. The antigens were analyzed for their B cell epitopic content using linear and conformational B cell epitope prediction tools. The final diagnostic antigen was designed by fusing the selected epitopic determinants form each antigen.

**Results:**

Given the conservancy results and B cell epitope predictions, the whole B2 antigen along with amino acids spanning 1–50, 1–30, and 30–81 regions of EPC1, B1 and B4 antigens were selected to design the final antigen. High surface accessibility (75%), protein stability, low free energy and high number of amino acids involved in B cell epitopes were desirable properties for the final antigen to interact with antibodies against CE.

**Conclusion:**

In silico design of such antigens is useful for better diagnosis of CE, decrease the cost and the time required for antigen design, while avoiding the ethical aspects of in vivo studies.

## Introduction

Cystic echinococcosis (CE), also known as hydatid disease, is a neglected deleterious helminthic disease. It is a type of zoonosis caused by the larval form (metacestode) of the tapeworm *Echinococcus granulosus* (*E. granulosus*) complex. Dogs and other canids are considered the definitive hosts of the *E. granulosus* domestic transmission cycle. However, ingestion of food and water sources contaminated with the parasite eggs excreted by definitive hosts could infect intermediate hosts. Sheep and other livestock are among the natural intermediate hosts of the parasite. Humans could become accidental or aberrant intermediate hosts by ingesting the eggs through direct contact with the definitive hosts or indirectly through contaminated food, water or soil. CE is still an outstanding health issue in the nomadic areas of Central and Middle Asia, Eastern Europe, Africa, Australia, South America and northwestern China (1, 2). An estimated 4 million human cases are globally infected with CE and 40 million humans are at risk of CE infection (3).

Once ingested, the eggs hatch in small intestine and onchospheres start to penetrate the intestinal wall into circulation. Liver and lung are the body organs that the hatched oncospheres choose to grow slowly in cysts filled with hydatid fluid. CE could lead to estimated mortality rates as high as 2%–4%, developing severe and life-threatening complications (4, 5). However, CE remains asymptomatic without discernable clinical manifestations until the infected organ is crowded with cysts larger than 7.5 cm in diameter (6, 7).

Due to the absence of pathognomonic signs in the early stages of the disease, the CE diagnosis remains with challenging issues. Early diagnosis of the disease would significantly contribute to the efficacy of the therapeutic strategies to cure CE. Therefore, circumventing diagnostic challenges would significantly reduce the morbidity and mortality rates associated with CE. Imaging scans including ultrasonography (US), computed tomography (CT), and magnetic resonance imaging (MRI) and serological tests like ELISA based methods are among the most used diagnostic approaches for CE detection. The results of diagnostic modalities based on imaging should be dealt with extra caution to distinguish between CE and alveolar echinococcosis (AE), cystic lesions, liver cirrhosis, and primary hepatocellular carcinoma (8, 9).

Moreover, confirming the larvae is difficult using imaging diagnosis. On the other hands, early detection of specific IgG antibodies in the human CE cases is the main serological method for CE diagnosis. This method could also be associated with insufficiencies like low sensitivity and specificity and poor prognostic value for follow-up (10).

Contemporary, recombinant antigens are widely used to detect various diseases including CE. This kind of CE detection tools would decrease the cross-reactions with other tapeworms and significantly increase the specificity and sensitivity of serological detection tests (11). The antigen B of the CE is composed of subsets with different molecular weights including 8, 16, 24 and 32 kDa. All of these subsets are complexes of 8 kDa subunit (12). Up to now, 5 subsets have been identified for 8 kDa subunit namely EgB8/1, EgB8/2, EgB8/3, EgB8/4 and EgB8/5 (13). Immunological properties of different subsets of antigen B are important for CE detection. Prior immunological studies about the subsets of antigen B are mainly focused on antigen B1 and B2, which were the first identified subsets of antigen B (11, 14). Moreover, other CE antigens have been used for its detection. The recombinant Epc1 antigen is among the antigens used for CE detection. Using recombinant Epc1 antigen would increase the specificity and sensitivity of CE detection tests (15).

Regarding the diagnostic challenges of CE detection, designing a novel diagnostic antigen would bring about significant advantages in fight against CE. In silico approach of designing novel diagnostic and therapeutic agents has long become an inevitable method in biological investigations. Exploiting these methods would minimize the need for arduous, costly and time-consuming empirical experiments (16, 17).

In the present study, we have used an integrated into silico approach to design a novel diagnostic antigen capable of CE detection. Various bioinformatics tools have been harnessed for structural and immunological analyses of EPC1 (protoscolex calcium binding protein), B2, B1 and B4 (subunits of AgB) antigens from *E. granulosus*. Ultimately, the most immunogenic domains of each antigen were used to design a final diagnostic antigen capable of the detection of CE infection.

## Materials and Methods

### Sequence analyses

This study was conducted at Tehran University of Medical Sciences, Tehran, Iran in 2018. The protein sequences for B2, EPC1, B1 and B4 antigens were collected from the NCBI protein database at (https://www.ncbi.nlm.nih.gov/protein). The sequences of the proteins were confirmed searching the UniProt database at http://uniprot.org/. The SignalP software at http://www.cbs.dtu.dk/services/SignalP/ was employed to analyze the sequences for existing signal peptides.

### Conservancy analyses

In order to determine the conserved regions of the B2, EPC1, B1 and B4 antigens, the protein sequence of each protein was used for BLAST search at https://blast.ncbi.nlm.nih.gov/Blast.cgi. BLAST search would find the sequences with highest similarity to the B2, EPC1, B1 and B4 sequences. The obtained sequences were used for Multiple Sequence Alignment analyses using Clustal W software at https://www.ebi.ac.uk/Tools/msa/clustalw2/.

### Secondary structure analyses

The secondary structures of the B2, EPC1, B1 and B4 antigens were predicted using SOPMA software at https://npsa-prabi.ibcp.fr/NPSA/npsa_sopma.html, PSIPRED at http://bioinf.cs.ucl.ac.uk/psipred/, PSSpred at https://zhanglab.ccmb.med.umich.edu/PSSpred/ and APSSP2 at http://crdd.osdd.net/raghava/apssp2/.

### 3D structure prediction

The 3D structure of the B2, EPC1, B1, and B4 antigens were predicted using homology modeling, threading and ab initio approaches. Homology modeling is the most reliable approach to predict the 3D structure of proteins. In this regard, a protein BLAST search against DPB database was done for each antigen to find suitable template structures for their homology modeling. The I-TASSER software at https://zhanglab.ccmb.med.umich.edu/I-TASSER/, MUSTER software at https://zhanglab.ccmb.med.umich.edu/I-TASSER/, LOMETS software at https://zhanglab.ccmb.med.umich.edu/LOMETS/ and QUARK software at https://zhanglab.ccmb.med.umich.edu/QUARK/ were used to predict the 3D structure of the antigens.

### Quality assessment and model refinement

The quality of the predicted models for each antigen was determined using QMEAN software at https://swissmodel.expasy.org/qmean/ and Prosa software at http://prosa.services.came.sbg.ac.at/. The selected models were fed as input for 3DRefine software at http://sysbio.rnet.missouri.edu/3Drefine/ for complete structural refinement. The low-quality regions of the refined models were remodeled using ModLoop software at https://modbase.compbio.ucsf.edu/modloop/. The amino acids 67–80, 54–70, 22–28 and 1–10 from the B2, EPC1, B1 and B4 antigens, respectively were selected for loop modeling.

### B cell epitope predictions

Various B cell epitope prediction software was invoked to analyze the 3D structures and the sequences of B2, EPC1, B1 and B4 antigens to identify their existing linear and conformational B cell epitopes. The linear B cell epitopes were predicted using Bepipred at http://tools.iedb.org/bcell/, BCpred at http://ailab.ist.psu.edu/bcpred/, LBtope at http://crdd.osdd.net/raghava/lbtope/, BcePred at http://crdd.osdd.net/raghava/bcepred/, and COBEpro at http://scratch.proteomics.ics.uci.edu/. The conformational B cell epitopes were predicted using ElliPro at http://tools.iedb.org/ellipro/, DiscoTope at http://tools.iedb.org/discotope/ and CBtope at http://crdd.osdd.net/raghava/cbtope/.

### Antigen design

In order to build the final antigen, the whole B2 antigen, 1–50 from EPC1, 1–30 from B1 and 30–81 from B4 were connected with a G4S linker (Glycine- Glycine- Glycine- Glycine-Serine). All possible orders of four antigens were checked to find the best antigen. The 3D structures of the built antigens were predicted using I-TASSER software. The free energy of each antigen was also calculated using SPDBV software. The sequences of the built antigens were assessed for existing B cell epitopes using the mentioned software.

### Physicochemical assessments and surface accessibility

The physicochemical properties of the final antigen were also determined. The ProtParam software at https://web.expasy.org/protparam/ was employed to determine the physicochemical properties of the final antigen. The surface accessibility of the final antigen amino acids was predicted using NetSurfP ver. 1.1 software at http://www.cbs.dtu.dk/services/NetSurfP/.

## Results

### Sequence retrieval

The protein sequences of B2, EPC1, B1 and B4 antigens were harvested from NCBI database under the accession numbers of AAC47169.1, AOY34842.1, AAD38373.1 and AAW78449.1, respectively. The sequences were confirmed by the sequences obtained from UniProt database. The signal peptide analyses indicated that only B2 (first 20 amino acids) and B1 (first 16 amino acids) had signal peptides sequences located at their N terminus end. The signal peptide sequences were omitted for following analyses.

### Sequence conservancy results

**Fig. 1:**
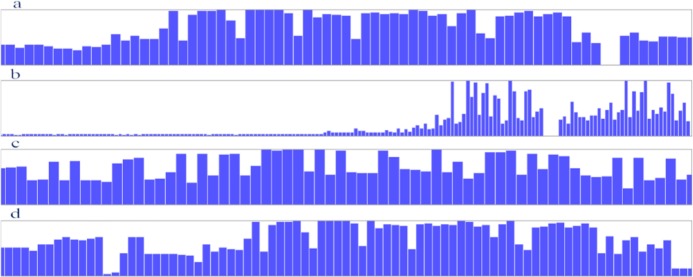
The conservancy results in sequences of the B2 (a), EPC1 (b), B1 (c) and B4 (d). The blue bars indicate the conservancy scale of the sequences. The higher the bars, the more conserved the sequence.

The BLAST search analyses returned a large number of similar sequences for each antigen. Multiple sequence alignment of the obtained sequences indicated that the sequence of B2 antigen is more conserve at the central region of the antigen. The sequence of EPC1 antigen has the lowest conservancy, the sequence of B1 antigen is more conserve at the central region of the antigen and the sequence of B4 antigen is more conserve at the central and C terminus region of the antigen (Fig. 1).

### Secondary structure prediction

**Fig. 2:**
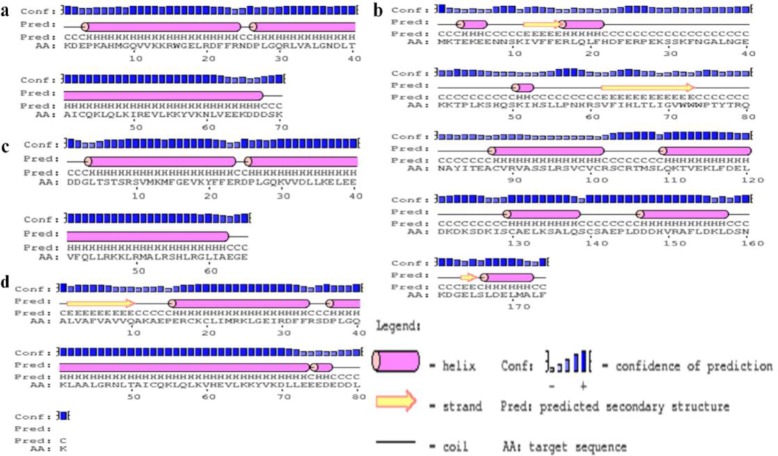
The secondary structures of the B2 (a), EPC1 (b), B1 (c) and B4 (d) sequences

The secondary structures of the B2, EPC1, B1 and B4 antigens were successfully predicted. Each antigen contains helixes, strands and coils. Figure 2 shows the secondary structures of the B2, EPC1, B1 and B4 antigens.

### 3D structure prediction

The protein BLAST search results indicated that there are no reliable template structures for homology modeling of the B2, EPC1, B1 and B4 antigens. Therefore, the software employing the threading and ab initio approaches or their combination was used for 3D structure predictions. All structure prediction software succeeded to model the structures of the antigens.

### Model assessment and refinement

**Fig. 3:**
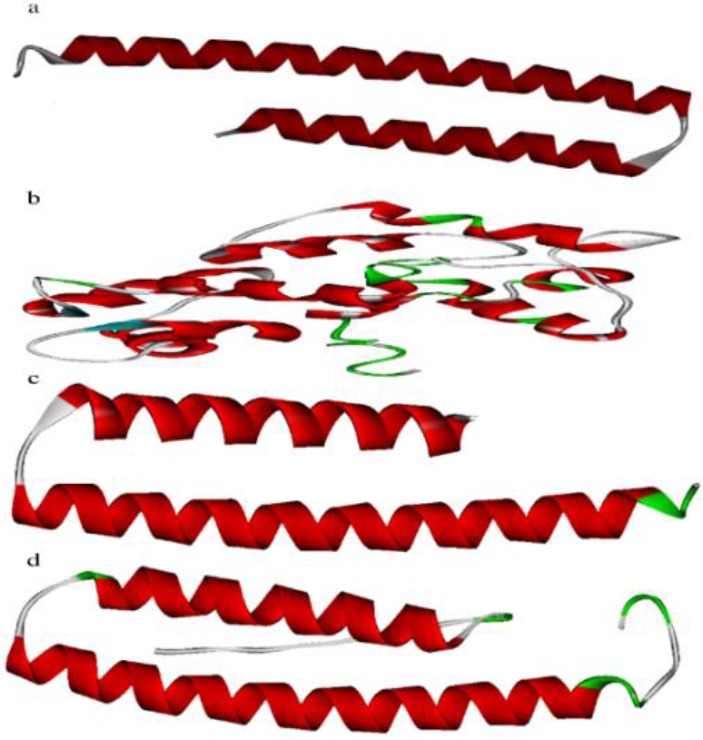
The 3D structures of the B2 (a), EPC1 (b), B1 (c) and B4 (d) sequences

The quality assessment of the predicted models indicated that the QUARK software managed to predict the best models for B2, B1 and B4, while the I-TASSER software predicted the best model for EPC1 antigen (Fig. 3). The model refinement and loop modeling analyses lead to improve model quality for all antigens, the QMEAN Z-scores are reported for each model, where the higher the Z-scores is capable of predicting the better model (Table 1).

**Table 1:** The quality of the predicted models before and after refinements listed for B2, EPC1, B1 and B4 antigens

***Epitope***	***Original model***	***3D refined model***	***Loop modeled model***
B2	−0.09	0.10	0.29
EPC1	−7.53	−4.94	−4.65
B1	−2.19	−1.09	−0.67
B4	−2.79	−0.92	−0.61

### Linear and conformational B cell epitopes

**Fig. 4:**
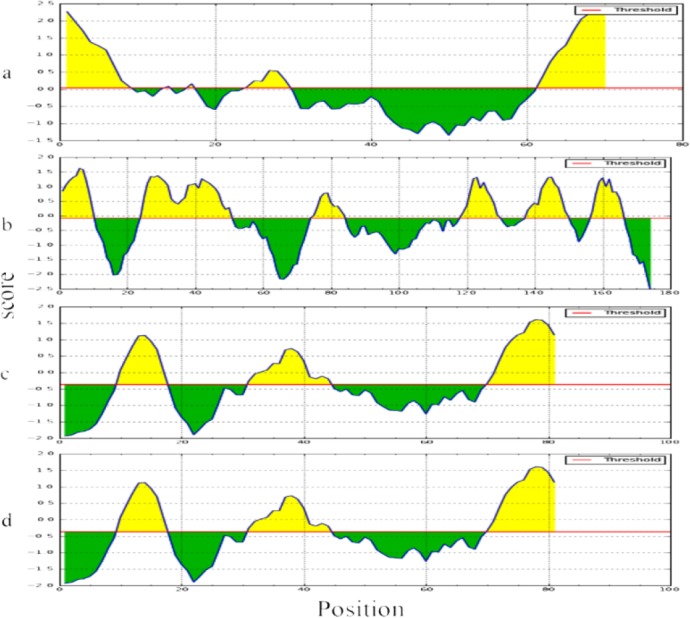
Linear B cell epitope prediction results. The figure illustrates the possible B cell epitopes within the sequence of B2 (a), EPC1 (b), B1 (c) and B4 (d), antigens. The regions above the threshold line which are in yellow are the regions with highest possibility of B cell epitopes.

**Fig. 5:**
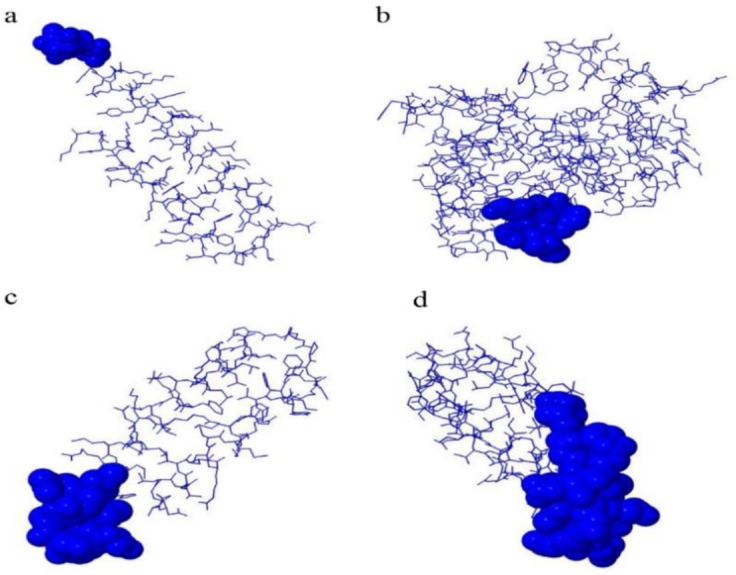
Conformational B cell epitope prediction results. The figure illustrates the possible conformational B cell epitopes within the sequence of B2 (a), EPC1 (b), B1 (c) and B4 (d), antigens. The epitopes with highest scores are depicted in spheres for each antigen.

The B cell epitope prediction results indicate that all of the antigens contain linear (Fig. 4) and conformation (Fig. 5) epitopes within their sequences and structures. Taken both kinds of the predicted epitopes into account, amino acids spanning 1–50, 1–30 and 30–81 regions of EPC1, B1 and B4 antigens are the regions with highest B cell epitope density. Moreover, the amino acids spanning 1–30 and 60–70 regions of B2 antigen are the regions with highest B cell epitope density.

### Final antigen design

**Fig. 6:**
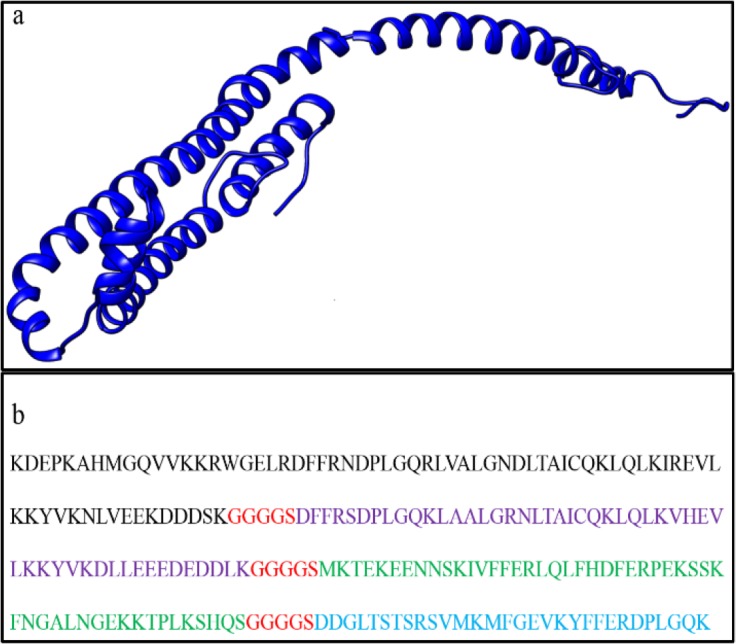
The structure and sequence of the finally designed antigen. The structure of the final antigen is presented in ribbon view (a). The sequence order of the final antigen is presented in color-keyed one letter amino acid (b) (Black is B2, purple is B4, green is EPC1, blue is B1 and red is the linker sequence).

All of the built sequences were successfully modeled and their free energy along with the number of their amino acids involved in B cell epitopes were determined. The antigen that had the highest number of exposed amino acids, lowest free energy and highest number of amino acids involved in B cell epitopes were selected as the final antigen. The sequence and structure of the final antigen are presented in Fig. 6.

### Physicochemical properties of final antigen

Assessing the physicochemical properties of the final antigen indicated that the designed antigen would be a 24 kDa antigen with isoelectric point (pI) of 8.87. Moreover, the final antigen was classified to be a stable protein. The surface accessibility analysis indicated that 77 out of 216 amino acids of the final antigen would be buried. This means that 75% of the amino acids are surface accessible.

## Discussion

Although CE could cause a severe chronic disease in human patients, WHO has announced CE as a neglected zoonotic disease (NZD) and a neglected tropical disease (NTD) (http://www.who.int/entity/neglected_diseases/en/, as accessed on 15 Sept 2017). Due to its asymptomatic nature during the early phase of infection, CE could impose numerous important negative economic effects, which are more tangible regarding expensive medical treatments for human cases and its large diffusion among livestock (18). Taken these facts into account, early diagnosis of CE is highly vital to avoid undesirable consequences. Therefore, using an integrative in silico approach, we have designed a novel diagnostic antigen most likely capable of circumventing the insufficiencies associated with pre-existing diagnostic moieties.

Clinical diagnosis of CE has mainly been practiced through the history and clinical signs, imaging scans and serological methods (19). However, serological methods have been highly preferred in the endemic regions of the disease due to lower cost, simplicity and availability of the tests. Despite the aforementioned advantages for serological methods of CE diagnosis, they suffer from low sensitivity and specificity (20). In order to overcome these drawbacks, designing an antigen encompassing the epitope determinants of more than one *E. granulosus* antigen would compensate for the diagnostic insufficiencies of each individual antigen.

B2, EPC1, B1 and B4 antigens were selected to design our diagnostic antigen. Antigen B of *E. granulosus* is one of the most abundant antigens of hydatid cyst fluid, widely studied for its diagnostic potentials (21, 22). This antigen comprises several related proteins codified by a multi-genic family composed of at least five genetic groups including antigen B1 to B5 (13, 23–25).

Recombinant antigen B is capable of improving the cross-reactivity rates, can be easily standardized and expressed in high volumes (14). Paralogous subunits B1, B2 and B4 are the main reactive subunits of antigen B family in sera detection and have been used for CE diagnoses (26). Cocktail subunits may improve the positive detection rate. Given these features, we have included all three B1, B2 and B4 subunits as a cocktail to improve the detection rates. Amongst these antigens, the whole sequence of the antigen B2 was included since it is one of the most extensively studied antigens for CE diagnosis (11, 14).

To avoid unwanted antigen size, only the epitopic determinants of the other antigen B subunits were encompassed within the design antigen. Smaller antigen size would provide the diagnostic antigen to have the epitopic regions with higher accessibility and less number of epitopes would be buried inaccessible for antibody interactions. The high number of exposed amino acids (about 75%) as predicted by the employed software indicated the validity of this approach. Moreover, the use of antigenic epitopes (instead of whole antigen sequences) would decrease the possibility for unwanted cross-reactivity against other pathogens, leading to increase in specificity and specificity (11).

Antigen 5 is one of the other *E. granulosus* antigens extensively studied for CE diagnosis. However, the cross-reactivity of this antigen has been reported with other parasites such as *E. multilocularis*, *E. vogeli*, and *Taenia solium* leading to reduction of the specificity of the antigen (27). Recombinant EpC1 is capable of increasing the diagnostic specificity of CE detection without decreasing the sensitivity rates, inserted in our final construct (15). Furthermore, performance of 2b2t+EPC1 fusion protein in ELISA showed promising results for serodiagnosis of human CE (28).

This property was of great interest for designing an antigen with high specificity and sensitivity. Selection of the epitopic determinants was done based on the density of both linear and conformational epitopes. In this regard, the structures of all antigens were predicted. The concordance between the predicted secondary and tertiary structures for all antigens indicated the accuracy of the predicted structures. Reliable 3D structures, in turn, would help to have better prediction for conformational B cell epitopes and selection of more suitable regions for final antigen. Variations within the sequences of analyzed antigens could reduce the accuracy of the performed diagnosis. The interaction between the finally designed antigen and the serum antibodies against *E. granulosus* antigens could fail due to the sequence variations of the infecting parasite. Therefore, we have considered the conservancy results to select the antigenic epitopes and the highly variable regions were avoided within the selected regions. This property would enhance the success rate and lower the false negative cases for the designed antigen. High surface accessibility, protein stability, low free energy and high number of amino acids involved in B cell epitopes were the predicted properties of the final antigen. These features were all in favor of a well-designed diagnostic antigen.

## Conclusion

In silico methods could be deemed as amenable alternative approaches to design diagnostic antigens. These methods would fairly decrease the cost and the time required for antigen design while avoiding the ethical aspects of in vivo studies. Herein, using an in silico approach, we have designed an antigen capable of sensitive and specific diagnosis of CE. A cocktail of epitopic determinants of B2, EPC1, B1 and B4 antigens were fused in a suitable order to design the final antigen. Assessing the properties of the final antigen indicated that this antigen would most likely be able to interact with antibodies against *E. granulosus*. However, experimental assessment of this antigen would bring better insights about the capability of the antigen for CE diagnosis.

## Ethical considerations

Ethical issues (Including plagiarism, informed consent, misconduct, data fabrication and/or falsification, double publication and/or submission, redundancy, etc.) have been completely observed by the authors.
